# The impact of a personalised action plan delivered at discharge to patients with COPD on readmissions: a pilot study

**DOI:** 10.1111/scs.12798

**Published:** 2019-12-21

**Authors:** Annette Hegelund, Ingrid Charlotte Andersen, Marianne N. Andersen, Uffe Bodtger

**Affiliations:** ^1^ Competence Center for Pulmonary Disease Department of Medicine Næstved and Slagelse Hospitals Næstved Denmark; ^2^ Department of Medicine Næstved and Slagelse Hospitals Odense Denmark; ^3^ Institute of Regional Health Research University of Southern Denmark Odense Denmark; ^4^ Department of Respiratory Medicine Næstved Hospital Odense Denmark

**Keywords:** COPD, personalised action plan, self‐management, COPD Assessment Test (CAT), discharge, cross‐sectorial, readmission, anxiety, depression, questionnaire

## Abstract

**Background:**

Self‐management interventions in COPD, including action plans, have the potential to increase quality of life and to reduce respiratory‐related hospitalisations. However, knowledge is still sparse of the effectiveness of a personally tailored action plan introduced at or right after discharge from hospital.

**Aim:**

This pilot study aimed to test whether a personalised, stepwise action plan supported with a short instruction provided at or postdischarge after an acute exacerbation in chronic obstructive pulmonary disease admission as an addition to usual care reduces readmissions and symptom burden, including anxiety and depression levels at 3‐month follow‐up.

**Methods:**

The study was carried out in a randomised controlled design with follow‐up after 3 months. In all, 75 participants were randomly assigned to either an intervention group that received an action plan, including the COPD Assessment Test (CAT), or to a control group that received usual care. The incidence of COPD‐related readmissions was measured as the primary outcome.

**Results:**

Compared to the control group, the action plan group significantly reduced the incidence of readmissions. The action plan group showed a trend towards a significant decrease in HADS‐depression, but none in HADS‐anxiety. Significant improvements in CAT scores were observed for the participants in the intervention group. Only inferior minor differences were found in use of inhalation therapy.

**Conclusions:**

A personally tailored action plan introduced at or postdischarge combined with follow‐up support is an effective self‐management tool to support recovery and to reduce unnecessary readmissions. In future follow‐up care, the healthcare professional must initiate the action plan at discharge and immediately after having the opportunity to follow the patient at home. This might require healthcare professionals working across healthcare sectors, who support patients until they have the needed confidence and competence in using the plan.

## Introduction

Chronic obstructive pulmonary disease (COPD) is a preventable and treatable condition characterised by dyspnoea, fixed airflow limitation, and is most often caused by increased airway inflammatory response to particle/gas exposure 1. COPD is associated with a substantial burden of illness and an increased risk of acute exacerbations of COPD (AECOPD), infections, heart failure, anxiety, depression, osteoporosis, diabetes, hospital admissions and death 2, 3. AECOPD is defined as being mild if the patient can manage them with changes in inhalation therapy, as moderate if treated by oral prednisolone and/or antibiotics by the general practitioner, and as severe if needing admission to hospital including day care 1.

Worldwide, AECOPD is a very common cause of emergency admission and there is a high readmission rate following discharge 4. The challenges related to unscheduled admissions as well as readmissions are considerable. In Denmark, the rate of admissions is slightly increasing and the rate of early readmissions within 30 days is about 19% 5. Repeated admissions for AECOPD are associated with anxiety and depression, reduced quality of life, loss of functions of daily living and an even higher risk of mortality 2, 6. Several factors affect decisions leading to admission: the severity of physical symptoms, the psychosocial distress, psychological stamina and the patient’s and relatives’ ability to respond adequately in the early phase of an AECOPD event 7. The public costs for COPD‐related admissions are increasing, in both the primary and secondary health sectors 8. Discharge from hospital after AECOPD is associated with anxiety and loss of security, and anxiety is associated with an increased risk of readmission 1, 6, 9. Consequently, growing international attention has been drawn to prevent early hospital readmissions to limit the physical deterioration and to contain healthcare costs 4. Although many initiatives have been implemented to improve quality of health care in recent years, reducing readmissions for AECOPD remains a problem that might require solutions that ensure a comprehensive and personalised prevention strategy for the individual COPD patient 4, 10.

In healthcare related to hospital discharge and follow‐up, it is essential to offer patients sufficient education and support to reinforce their self‐management to acquire the necessary skills to manage and cope on a day‐to‐day basis with their disease, assume greater responsibility for healthcare decision and maintain behaviours that improve their well‐being. The self‐management support patients to recognise and initiate early treatment to avoid progression from mild AECOPD to moderate or severe AECOPD and thereby preventing hospital admissions. Most recent literature reviews have concluded that multicomponent self‐management interventions including an COPD action plan (AP) combined with brief education reduce respiratory‐related hospital admissions and improve quality of life 10, 11, 12, 13. AP encourages patients to identify daily variations in symptoms, and to take appropriate actions, if needed, that is change medication regime or contact a healthcare professional 14. However, the onset of an AECOPD can be difficult to identify and as leading researchers within primary and respiratory care Pinnock *et al.* argue, the challenge is that APs often do not fully reflect these difficulties 15. According to included studies in the Cochrane reviews within the field, the research on APs has mostly been performed in outpatient settings in a stable phase of disease 12, 13. Nevertheless, qualitative work has highlighted that not all patients have the ability to attend outpatient education after discharge due to an overall vulnerability, impairing the difficulty accepting the chronic disease, the understanding of being frail or the altered life situation 16. Besides, experiences of difficult access and contact to healthcare professionals after discharge can dominate and complicate COPD self‐management resulting in doubt and hopelessness over time 17.

Therefore, we aimed at testing whether a simple, written, individual, stepwise AP supported with a short patient‐involved instruction provided at or postdischarge after an AECOPD‐related admission as an addition to usual care reduces readmissions and symptom burden including anxiety and depression levels at 3‐month follow‐up.

## Methods

### Study design

The pilot study was a prospective, 1:1 randomised, clinical, un‐blinded, complex intervention study delivering an AP or usual care. The study was designed as a pilot test in order to judge the feasibility whether or not such an intervention delivered during an AECOPD‐related admission was relevant and sustainable. All data were collected prospectively, except data on number of admissions during a 3‐month period before and after index admission which were collected retrospectively.

### Outcome measures

#### Primary outcome measure

Incidence of COPD‐related readmissions during three months after discharge from index admission (i.e. when the patient was included in the study).

#### Secondary outcome measures

Differences in Hospital Anxiety and Depression score (HADS) including subscores 18, COPD Assessment Test (CAT) 19, drug inhalation therapy, use of long‐term oxygen therapy (LTOT), and home nebulisers at inclusion (baseline) and at follow‐up, including changes in number of therapeutic changes.

### Participants

Patients were eligible if known doctor‐diagnosed COPD, admitted to the department of respiratory medicine at Næstved or Slagelse Hospital with AECOPD between August 2016 and February 2017, had at least one prior admission for AECOPD in the preceding 3 months, and had passed the acute phase on their inpatient care. Exclusion criteria were acute respiratory failure, poor cognitive function, expected survival shorter than 3 months, and severe physical or mental impairment due to other diseases than COPD.

### Patient selection, randomisation and data collection

Participants were recruited between August 2016 and forward until 100 patients were identified. Last patient was included on 22 February 2017.

Randomisation was conducted by two respiratory‐educated study nurses, who visited each respiratory medicine unit twice weekly (Næstved: Mondays and Thursdays; Slagelse: Tuesdays and Fridays). At each visit, potentially eligible patient was allocated a unique identification number before oral and written informed consent was obtained. Participants with odd identification numbers were randomised to intervention, and those with even numbers to control. At baseline, all participants completed the Hospital and Anxiety Depression Scale (HADS) and COPD Assessment Test (CAT) and reported number of persons in household, assistance at home, and comorbidity. The burden of comorbidity was classified by use of the Charlson index of comorbidity 20.

Data on number of hospital admission, COPD medication, lung function, gender, age, smoking status, use of LTOT, and nebuliser were extracted from electronic medical records. At 3‐month follow‐up, all participants were contacted to repeat completion of both CAT and HADS.

### Questionnaires

To assess the burden of symptoms, the COPD Assessment Test (CAT) was applied. CAT is a patient‐completed instrument to reflect the impact of COPD on patients’ health status. CAT provides a simple and reliable measure of health status in COPD to aid assessment of patients, promote communication between patients and clinicians, and enabling a common understanding of the severity and impact of the patient’s disease 19. Intensity of symptoms is measured on a rating scale ranging from 0 to 5 giving CAT a max score of 40, and a change of two units is accepted as minimum clinically significant change 21.

Hospital and Anxiety Depression Scale (HADS) is used to measure symptoms of anxiety and depression. Both domains consist of seven statements on emotions or emotional situations. Patients express their agreements with the statements on a scale from 0 to 3, which leads to a maximum score of 21 points for each domain. Scores between 8 and 11 per domain are suggestive of the precedence of the mood disorder, and scores = 11 or more indicate a probable presence of anxiety/depression symptoms 18.

### Control group

The participants in the control group received usual care and treatment according to GOLD guidelines 1 and the Regional Disease Management Guidelines 22, 23 from the multiprofessional staff employed at the respiratory medicine units (doctors, nurses, physiotherapist, occupational therapists, dieticians etc.). Usual treatment and care consisted of both pharmacological and nonpharmacological care according to most recent evidence‐based guidelines on COPD 1 comprising information of, that is, vaccination, inhalation techniques, optimising medication, sputum mobilisation, breathe control, exercise, nutritional aspects, smoking (cessation), management of exacerbations and psychosocial support provided on an as needed basis to the decision of the responsible healthcare professionals. Outpatient clinics, general practitioners (GP) and municipality are involved in the follow‐up on medical treatment, COPD rehabilitation, homecare and/or home nursing after discharged if needed. The participants in the control group were—after study termination—offered an AP identical to that received by the intervention group.

### Intervention

In addition to usual care, the participants received a comprehensive intervention under the index admission consisting of partly a personalised stepwise written AP, partly a self‐management dialogue including short instruction and the possibility for subsequent support. The approach to the intervention was coaching and structured, but still personalised and multicomponent in accordance with the international recommendations 24. The AP, used in this study, was a three‐coloured plan to improve self‐management and treatment for patients with COPD supplied with CAT guiding to the right step for action.

#### Dialogue and instruction

The participants were individually introduced to and shortly instructed to the AP. The dialogue was based on opening questions like: ‘What matters to you?’, ‘How do you manage changes in your daily condition?’ ‘What would you like to be able to manage?’ On behalf of the participants’ identified needs and motivation, instructions to handle symptoms were formulated in the participants’ own language and written in the AP. The final AP was completed either at discharge or as telephone‐based care or as a home visit within the following three weeks and made available to the participant as a personal paper sheet. The spouse participated as well by the choice of the patient. Elapsed time to the individual instruction merged 1‐2 hours. If the study nurse, during the dialogue with the participants, observed specific needs for treatment or care beyond the intension of the intervention, she was ethically obliged to inform the staff at the respiratory medicine units.

#### Action plan

The AP was a one‐side A4 paper sheet, and a map illustrated in green, yellow and red colour according to the participants’ perceived condition of daily function or symptoms clarified in CAT 19 such as shortness of breath, coughing, sputum and physical abilities (*e.g.* ‘what do I normally manage, when I am in this condition’) with suggestions of specific nonpharmacologic and pharmacologic actions. The green step illustrated the stable state concerning condition.

**Figure 1:**
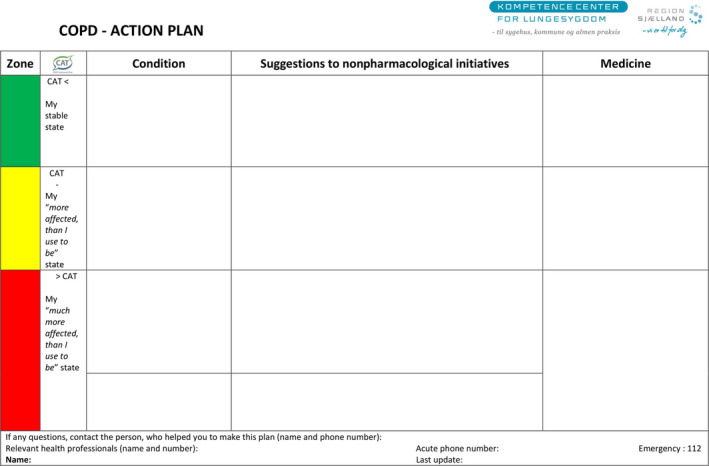
COPD action plan.

Yellow step illustrated increased symptoms and actions in the ‘affected more than usual’ state, whereas the red step in the ‘much more affected than usual’ state. The three steps were separated in four columns concerning CAT score, the condition, suggestions to nonpharmacologic initiatives and medicine (see Fig. 1) . The pharmacologic and nonpharmacologic treatments were described in order to improve the participants’ own ability to take actions in different situations compared to the usual everyday life and condition with COPD.

In the initial process of tailoring the AP, the study nurse instructed each participant to measure and self‐report three CAT scores within a week. Based on these CAT scores and the experienced condition, the study nurse encouraged the participant to define the content of the green, yellow or red step, respectively.

In a dialogue, professional instructions were provided to each step according to nonpharmacologic initiatives adjusted to the participants’ individual capability and states. They were instructed in taking actions related to each step, according to observation of sputum, breathing exercises, physical training, daily activities, food and nutrition, and recommended to use CAT once a month in the green step and more frequently, if they perceived themselves affected more than usual. In case of> 3 points increase in CAT, participants proceeded to next step in their AP.

Furthermore, the management of and adherence to inhalation medicine, including technique, was checked and corrected if needed. Participants were instructed how to change pharmacological treatment related to each step in the AP, for example increasing use of short‐acting β2‐agonist (SABA) before activity or if needed. If antibiotics and corticoid steroid were prescribed from the GP in advance as a self‐administered rescue kit, they were added to the yellow step. Phone numbers to relevant healthcare professionals in each step are cleared.

Participants were invited to contact the study nurse by telephone for AP advice during the 3‐month period.

### Ethics

Approval from the Research Ethics Committee system was not required as no biological material was obtained during the study. Data collection was approved by the (blinded for review).

### Statistics

No formal power calculation was performed prior to study onset, as there are no defined minimally clinical relevant difference for number of admissions. Pragmatically, we considered any intergroup difference of low clinical importance if it was not demonstrated in a cohort of 100 patients, that is, 50 patients in each study arm, corresponding to power (1‐β) of 90%, α 0.05, difference 20%, and standard deviation 30%.

Data were analysed using statistical software (SPSS 21.0, IBM, Chicago, IL, USA). Discrete data were presented as median (range or interquartile range, IQR), and categorical data as number, n (%). Between‐group differences were analysed for statistical significance using Mann–Whitney *U* test (discrete data) or chi‐square test (categorical data). Intragroup differences were analysed using Wilcoxon’s signed rank test (discrete data). Significance was reached at p < 0.05.

## Results

### Baseline characteristics

**Figure 2:**
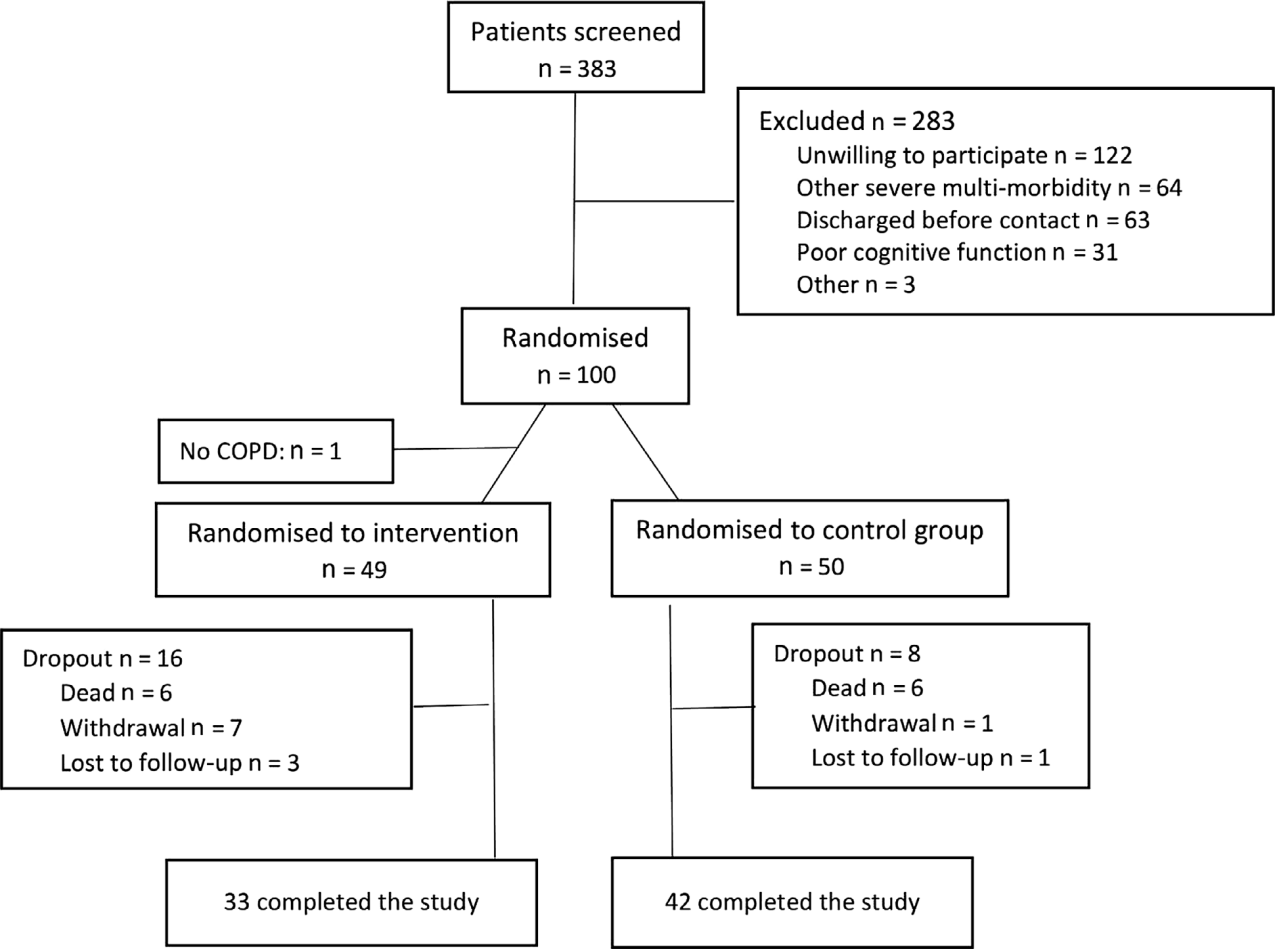
Consort diagram depicting flow of patient through the study.

Figure 2 shows that we randomised 100 patients to either AP or usual care: median age 73 years (range 45–89), GOLD D 45%, females 58%, living alone 48%, having council home help 37%, and having 1 (0–5; interquartile range 1–2) hospital admissions due to acute exacerbation in COPD within the last 3 months prior to the index admission. One case in the intervention group did not have COPD and was deleted from all analyses. Overall dropout was 16 (32%) in AP versus 8 (16%) in control group (p = 0.06) including six patients who died during the study (between‐group difference: p = 1.0). The higher dropout rate in the intervention group (although not statistically significant) was due to severe physical or mental impairment in other diseases than COPD. None in the AP group used the offer to contact the study nurse by telephone for AP advice during the 3‐month period.

Comparing baseline characteristics between groups revealed no significant differences (Table 1).

**Table 1:** Differences between groups (intention‐to‐treat cohort)

	Intervention group n = 49	Control group n = 50	*p*‐value
Female, n (%)	30 (61)	28 (57)	ns.
Age, median (interquartile range)	73 (67‐81)	72 (67‐78)	ns.
Never/former/current smoker, n^a^	2/38/8	2/38/9	ns.
Nebuliser at home, n (%)	5 (10)	12 (24)	0.07
Long‐term oxygen therapy, n (%)	8 (16)	9 (18)	ns.
Living alone, n (%)	19 (39)	28 (57)	0.08
Aid in home, n (%)	19 (39)	18 (37)	ns.
GOLD D, n (%)	35 (73)	29 (59)	ns.
COPD drug Inhalers at index admission			
LABA + LAMA+ICS, n (%)	32 (65)	30 (60)	ns.
LABA + LAMA, n (%)	5 (10)	3 (6)	
LABA or LAMA, n (%)	0	4 (8)	
ICS/ICS + LABA/ICS + LAMA, n (%)	10 (20)	8 (16)	
No controller, n (%)	2 (4)	5 (10)	
Charlson’s comorbidity index^b^, ^c^, median (interquartile range)	6 (5‐8)	6 (5‐8)	ns.
FEV1 % expected, median (range)	28 (19‐49)	41 (26‐57)	ns.

GOLD, Classification of COPD 1; LABA, long‐acting β2‐agonists;LAMA, long‐acting muscarin antagonists ICS, inhaled corticosteroids; FEV1: forced expiratory volume in one second 1; ns. = insignificant values> 0.

a Unknown smoking history, n = 3.

b Including COPD diagnosis.

c Charlson’s comorbidity Index 20.

### Readmissions for COPD during follow‐up (Primary outcome)

Table 2 shows that we observed significantly fewer readmissions during follow‐up in the AP group, but no difference in admissions in the 3 months prior to index admission.

**Table 2:** Differences in primary and secondary endpoints (intention‐to‐treat cohort)

	Intervention group n = 49	Control group n = 50	Intergroup difference p‐value
Number of admissions, median (IQ range)			
3 months before index admission	1 (1‐2)	1 (1‐2)	ns.
3 months after index admission	1 (0‐1)*	1 (1‐2) ^ns^	<0.01
Patients with 0 admissions, n (%)			
3 months before index admission	2 (4)	0 (0)	ns.
3 months after index admission	23 (48%)	0 (0)	<0.0001
COPD Assessment Test (CAT)^a^, median (IQ range)			
Baseline	23 (18‐27)	23 (18‐29)	ns.
Follow‐up	15 (11‐20)*	19 (12‐28)*	<0.05
Hospital Anxiety and Depression Scale (HADS)^b^			
Baseline	14 (8‐23)	15 (7‐22)	ns.
Follow‐up	6 (4‐12)*	9 (4‐18)*	ns.
HADS‐anxiety, median (IQ range)			
Baseline	7 (4‐13)	8 (3‐13)	ns.
Follow‐up	3 (1‐7)*	5 (2‐8)*	ns.
HADS‐depression, median (IQ range)			
Baseline	7 (4‐11)	6 (3‐11)	ns.
Follow‐up	4 (2‐7)*	5 (2‐10)^ns^	ns.

ns. = insignificant values> 0.1.

a COPD Assessment Test (CAT) 19

b Hospital Anxiety and Depression Scale (HADS) 18.

* p < 0.05 for intragroup differences.

The number of patients with no readmission in the intention‐to‐treat cohort was 23 (48%) in the AP group *vs*. 0 in the control group (p < 0.00001, *Fisher’s* exact test). In the per‐protocol group, the numbers in the the AP group were 17 (52%) respectively 0 in the control group (p < 0.00001, *Fisher's* exact test).

We found no significant relationship between no readmission and sex, age (<70 vs. ≥70 years), GOLD class (C *vs.* D), or household members (living alone *vs*. not alone). Table 3 shows minor differences between groups in use of inhalation therapy, home nebuliser use and LTOT at admission and at discharge in the per‐protocol group. However, in the intension‐to‐treat group, significantly more participants in the intervention group than in the control group were prescribed LTOT or home nebuliser (8 vs. 1, p < 0.05; none started on both). Onset of LTOT or home nebuliser therapy was not associated with reduced number of readmissions.

**Table 3:** Per‐protocol analyses of differences in use of medication, long‐term oxygen therapy (LTOT) and access to nebuliser at home between groups at recruitment and at discharge from index admission. For the intervention group, this corresponds to before and after education in action plan

	Intervention n = 33	Control n = 42	p‐value
SABA, admission, n (%)	32 (97%)	35 (83%)	0.07
SABA, discharge, n (%)	33 (100%)	35 (83%)	<0.05
LABA/LAMA, admission, n (%)	29 (88%)	28 (67%)	<0.05
LABA/LAMA, discharge, n (%)	29 (88%)	31 (74%)	ns
ICS/LABA/LAMA, admission, n (%)	27 (82%)	27 (64%)	ns
ICS/LABA/LAMA, discharge, n (%)	25 (76%)	25 (62%)	ns
Drug changes, n (%)	4 (12%)	10 (24%)	ns
LTOT, admission, n (%)	5 (15%)	5 (12%)	ns
LTOT, discharge, n (%)	8 (24%)	6 (14%)	ns
Nebuliser, admission, n (%)	4 (12%)	10 (24%)	ns
Nebuliser, discharge, n (%)	4 (12%)	10 (24%)	ns
Onset of LTOT or nebuliser, n (%)	3 (9%)	1 (2%)	ns

ns = insignificant values> 0.1; all in‐group comparison before vs after action plan: p> 0.3 (Wilcoxon’s test). ICS, inhaled corticosteroids; LABA, long‐acting β2‐agonists; LAMA, long‐acting muscarin antagonists; LTOT: long‐term oxygen therapy; SABA, short‐acting β2‐agonist.

### Symptoms (Secondary outcomes)

Table 2 shows that CAT scores did not differ between groups at baseline, but differed significantly at follow‐up (p < 0.05). Median CAT scores decreased significantly in both groups with a median decrease of 5 (IQR 0‐13) in AP group (p < 0.00001), and 2 (IQR −2‐8) in control group (p < 0.05).

### Anxiety and depression

We found no significant differences between groups in any HADS score at baseline or at follow‐up (Table 2). At study entrance, the overall HADS score was 14 (IQR 7‐22), with HADS‐A (anxiety) 8 (IQR 4‐13) and HADS‐D (depression) 6 (IQR 3‐10). Table 2 shows that total HADS and HADS‐A scores decreased significantly in both groups from baseline to follow‐up, but HADS‐D decreased significantly in the AP group only (Wilcoxon signed rank test, all analyses: p < 0.01). Including only patients completing the study (per‐protocol analyses) showed consistent findings. We did not find any significant association between reduction in HADS total or subscores in subgroup analyses (gender, age cut‐off ≥ 70, GOLD class, living single or not) (data not shown).

## Discussion

In the current pilot study, we demonstrated that a personalised AP provided at or postdischarge after an admission for AECOPD is feasible and significantly reduced number of readmissions in the following months. This adds to the evidence of action plans as being an important therapeutic intervention in COPD 12, 13. However, as stated by Lenferink *et al*. in their Cochrane review, the effectiveness of AP is not completely clear 12. Through our pilot study, we achieved valuable assessment of the effectiveness of an AP in which we payed specific attention to the personal difficulties of the participants in identifying and acting appropriately on an AECOPD at the time after discharge. In the following, we discuss some central mechanisms behind the positive outcome of our AP.

In qualitative research, patients’ recognition of COPD‐related worsening and performance of self‐management actions has been identified as the two most important self‐management skills 25. Generic factors as heterogeneity of exacerbations and habituation to symptoms are known to influence recognition of exacerbation and self‐management actions and to guide rational self‐management strategies 25, 26. In an interview study, COPD patients reported to use a combination of measurable ‘visible’ symptoms, like cough, sputum production, cold symptoms and functional limitations and ‘invisible’ symptoms, as soreness, tightness or heaviness of chest, lack of energy and ‘body‐knowing’ to identify and manage exacerbations, according to symptoms that had the most impact on their well‐being 27. Our AP implies the possibility of frequent measurements of CAT to support awareness and recognition of both visible and invisible changes in COPD‐related symptoms. The CAT score is not developed for this purpose yet, but appears to be a useful tool in AP for patients to point out that even small, immediate invisible changes can be identified and indicate needed actions to be taken. Previously, in a similar intervention study using an AP as a component, Trappenburg *et al.* employed the measurement tool, Clinical COPD Questionnaire (CCQ) in their AP every third day to measure the longitudinal course of the disease‐related health status supplied with diary cards 26. As recommended in GOLD, we chose CAT as the primary validated measurement tool to predict future AECOPD, even CCQ is mentioned as an alternative 1. Studies comparing CAT and CCQ show similar psychometric properties with a slight advantage for CCQ based mainly on patients’ preferences 28. However, CAT appears to predict future exacerbations events better and has a slightly shorter average time for questionnaire completion 29. As intended and tested in our pilot, the CAT appeared to be a useful tool. Thus, our results showed a significant decrease in CAT between the AP group and control group, when the participants had opportunity to use CAT as a measurement tool to guide them to find the right step in the AP showing them how to take appropriate actions.

Initiating an AP at or postdischarge differs from several studies, especially on the pharmacological treatment 26, 30, 31. In our intervention, the participants were not guided to initiate oral corticosteroid and/antibiotics at home, unless it was a part of their usual treatment plan. The intervention and control groups differed only slightly in changes in COPD therapy optimisation of guideline‐based medication in the AP group, during index admission (Table 3), except concerning initiation of LTOT or home nebuliser therapy (in 8 patients in intervention group *vs* 1 in control group, p < 0.05). Thus, it is likely that delivering an AP during admission may support the tailoring of personalised COPD therapy concerning both drugs and devices 1. Yet, drug or device changes seem not to be fully explain the observed reduction in COPD‐related admissions during follow‐up.

In our intervention, the same study nurse introduced the AP and followed up after discharge. In the intervention tested and evaluated by Trappenburg *et al*., they found that addition of a case‐manager, as a key component to the AP, could decrease the impact of exacerbations on health status, decrease symptom intensity of exacerbations and facilitate recovery of exacerbations 26. The case‐manager role is, according to other research, crucial when performing self‐management strategies in AP, pointing out that the interaction between case‐manager and patient often weighs more than the content itself 32. The case‐manager needs strong communicative skills, ability to assess patients’ learning outcomes and efficient use of teaching tools 32. The assistance of a possibility to telephone a case‐manager in questions to AP help to improve patients’ health and well‐being 33. Previously, multicomponent self‐management programs including case management have shown to improve patient outcomes and to reduce use of healthcare services 11. Thus, it seems that ongoing collaboration with healthcare professionals when introducing a tool as an AP can contribute to accelerate recovery and as seen in our intervention to reduce readmissions within three months.

Our study indicates that neither more guideline‐based medication nor reduction in anxiety or depression explained the observed reduction in readmission rate. However, patient‐reported depression, but not anxiety, decreased significantly in the AP group. This is to our knowledge not described in other studies. Depression is a common reaction to COPD and often developed in an ongoing vicious cycle between low mood, physical health problems and reduced activity. Heslop argues that enhancing nonpharmacological treatments as education and counselling to increase self‐management can be an important brick to break this vicious cycle 34. Maybe a small intervention as a personalised AP can contribute to break the cycle. To understand the mechanisms behind the effects of the AP further, we recommend this to be integrated in future studies of efficiency of self‐management interventions.

Our study has various limitations. We performed a small, two‐centre study with limited power, and we screened almost 400 patients to recruit 100 patients, and thus, our patients represent a selected group. This hampers external validity, so our results may not be reproduced in other COPD cohorts. Furthermore, by chance our groups were not numerically comparable as the control group had higher numbers of patients living alone, having home nebulisers, and having better lung function tests. We did not find a positive association between these parameters and our primary endpoint (perhaps due to the small sample size) but may be important to consider, since, for example, living alone is positively associated with poorer health outcomes, including anxiety and depression; thus, the relationship does point in different directions 35. Future studies will show whether the impact of AP during admission should be tailored according to whether the patient is living alone or not. Cohabiting relatives can be of significance to achieve the positive impact of the intervention as shown in our study, since family members often play an essential role in helping COPD patients to resume or strengthen their self‐management after a hospitalisation 17.

Of concern, 122 patients were not interested to participate in the study, and we lost almost 1/3 of patient in the intervention group. This suggests that the concept of AP may not be attractive to a substantial subgroup of the patients. We did not explore underlying causes for this aversion, but we speculate that the patients may find it relevant but too demanding, and other may consider it irrelevant or even an intrusion into their own sphere of responsibility? Qualitative research has highlighted how COPD patients struggle to self‐manage after discharge from an AECOPD and if not properly supported, the demand to take over a greater level of responsibility for their own care can place an additional burden on them 36. Another challenge is that clinical APs rarely consider the impact of multi‐morbidity 15. Thus, providing self‐management support, including AP, is a complex intervention that should integrate patients’ beliefs and preferences.

We had full access to medical journals for the entire region including data on admissions or emergency care visits at neighbouring and nearest five hospitals. We did not explore other healthcare contacts such as general practitioner or extra‐regional hospitals, but in Denmark, severe AECOPD will lead to admission at the local hospital, as there are no community clinics or relevant intermediate services. However, we showed that introducing AP during hospitalisation for frequent AECOPD is feasible and efficacious in reducing COPD‐related readmissions. Based on our pilot study, the findings need to be reproduced in other cohorts and healthcare settings, such as whether a personalised tailored AP with alleviating instructions could be appropriate to implement in a palliative care context. Furthermore, there is a need for personal adaptation of when and how to provide AP.

Valuable information could be obtained by future studies on patients’ perception of AP including those patients who do not happily accept the AP invitation but refuse of one reason or another.

## Conclusion

This pilot study has shown that a personalised written AP provided at or postdischarge after an admission for AECOPD, and based on a person‐centred self‐management approach, is able to reduce the number of readmissions significantly in the following three months. The AP had a significant positive effect on the feeling of depression compared to the control group. The use of CAT seemed to be a useful tool in the written AP to support recognising even small changes in COPD‐related symptoms, and the consciousness to take appropriate actions according to the AP. The opportunity for early and ongoing collaboration with a healthcare professional, when introducing a tool as an AP, can be important to accelerate recovery to reduce readmissions after AECOPD.

### Relevance for clinical practice

In addition to respiratory professional knowledge and practice, healthcare professionals need strong communicative and health‐educational skills. To improve clinical practice related to self‐management support in COPD, healthcare professional may introduce a personalised AP during hospital admission for AECOPD and supplement with a telephone‐based or a home‐visit follow‐up to complete the AP. Healthcare professionals working across healthcare sectors, that is a cross‐sectorial case‐manager, may in the future be central to offer a trusting and patient‐centred support to achieve patients’ confidence and competence in the AP.

## Author contributions

AH, MNA and UB involved in study design; MNA and UB involved in data collection; and AH, ICA, MNA and UB involved in analysis and manuscript preparation.

## Ethical approval

The study was approved by the Danish Data Protection Agency (ID no. 18‐000315/2016‐076). In accordance with Danish law, no further ethical approval was required.

## Funding

The study was funded by Department of Medicine 1, Næstved, Slagelse and Ringsted Hospitals, Denmark.
